# Amino Acid Supplementation as a Biostimulant in Medical Cannabis (*Cannabis sativa* L.) Plant Nutrition

**DOI:** 10.3389/fpls.2022.868350

**Published:** 2022-03-31

**Authors:** Matěj Malík, Jiří Velechovský, Lukáš Praus, Anežka Janatová, Zdeňka Kahánková, Pavel Klouček, Pavel Tlustoš

**Affiliations:** ^1^Department of Agroenvironmental Chemistry and Plant Nutrition, Faculty of Agrobiology, Food and Natural Resources, Czech University of Life Sciences Prague, Prague, Czechia; ^2^Department of Food Science, Faculty of Agrobiology, Food and Natural Resources, Czech University of Life Sciences Prague, Prague, Czechia

**Keywords:** medical cannabis, phytocannabinoids, amino acids, *Cannabis sativa* L., terpenoids, biostimulant, hydroponics

## Abstract

There is growing evidence to support the involvement of nutrients and biostimulants in plant secondary metabolism. Therefore, this study evaluated the potential of amino acid-based supplements that can influence different hydroponic nutrient cycles (systems) to enhance the cannabinoid and terpene profiles of medical cannabis plants. The results demonstrate that amino acid biostimulation significantly affected ion levels in different plant tissues (the “ionome”), increasing nitrogen and sulfur content but reducing calcium and iron content in both nutrient cycles. A significantly higher accumulation of nitrogen and sulfur was observed during the recirculation cycle, but the calcium level was lower in the whole plant. Medical cannabis plants in the drain-to-waste cycle matured 4 weeks earlier, but at the expense of a 196% lower maximum tetrahydrocannabinolic acid yield from flowers and a significantly lower concentration of monoterpene compounds than in the recirculation cycle. The amino acid treatments reduced the cannabinolic acid content in flowers by 44% compared to control in both nutritional cycles and increased the monoterpene content (limonene) up to 81% in the recirculation cycle and up to 123% in the drain-to-waste cycle; β-myrcene content was increased up to 139% in the recirculation cycle and up to 167% in the drain-to-waste cycle. Our results suggest that amino acid biostimulant supplements may help standardize the content of secondary metabolites in medical cannabis. Further experiments are needed to identify the optimal nutrient dosage and method of administration for various cannabis chemotypes grown in different media.

## Introduction

Medical cannabis research has developed dramatically in recent years (Grotenhermen and Muller-Vahl, 2012). The use of these plants in healthcare and pharmaceutics places rigorous demands on the growing environment for optimal production of the desired active compounds (Potter, 2014). For these reasons, and because it is now legal, many growers have opted to use indoor facilities as a more efficient way to grow medical cannabis, a method used mainly by illegal growers until recently (Drugs and Crime, 2009). Consequently, indoor cultivation has become more sophisticated with automated lighting, ventilation, and irrigation systems being commonly in use. It can be implemented in several ways, but always comes down to two basic methods—cultivation in soil substrates or hydroponically. The nutrients are dissolved in the irrigation water, or already fertilized soil substrates can be used. Hydroponics is currently one of the fastest developing methods in the horticultural industry (Vanhove et al., 2011) and cannabis growers have already started to use it extensively. In hydroponic cultivation the nutrients are supplied in the form of an aqueous solution directly in contact with the plant’s root system. Thanks to the possibility of year-round growth in a controlled environment, this method has the potential to produce high yields of homogeneous plant material of excellent quality (Bouchard and Dion, 2009).

At present, basic research information about regulating the biosynthesis of secondary metabolites of medical cannabis is lacking because of legal restrictions in most countries (Aguilar et al., 2018). With respect to the internal and external factors influencing the secondary metabolite content and spectrum of cannabinoids, the main determining internal factor is the genetics of *Cannabis sativa* L. subsp. *sativa* and subsp. *indica* (Janatová et al., 2018; Mcpartland, 2018). This directly impacts the chemotype, habitus, cannabinoid, and terpene profile of the cultivated cannabis plant (Aizpurua-Olaizola et al., 2016). However, the genetics and the plant phenotypes are strongly influenced by external factors, with growing conditions playing a crucial role in productivity and quality. The main external parameters include light (Danziger and Bernstein, 2021), irrigation (Caplan et al., 2019), carbon dioxide concentration (Chandra et al., 2011), and nutrition (Malík et al., 2021). Nutrients play a central role in many aspects of plant metabolism. There is a wealth of experimental evidence to support the effects of nutrients, especially nitrogen (Saloner and Bernstein, 2021), phosphorus (Shiponi and Bernstein, 2021a), and potassium (Yep et al., 2021), on secondary metabolites of medical cannabis plants. The cannabinoid and terpene profile of medical cannabis can be influenced by the concentration and ratio of these major nutrients (Caplan et al., 2017a; Bernstein et al., 2019). Although emphasis is placed on the availability of sufficient amounts of these major plant nutrients, the potential effects of micronutrients and plant biostimulants must also be considered (Bernstein et al., 2019).

Several studies have used protein hydrolysates and amino acids (AAs) as plant biostimulants. The mechanism of their action on plants is thought to involve modulating nitrogen absorption and assimilation by regulating the enzymes and structural proteins involved in these processes. AA biostimulants also affect nitrogen uptake by the roots through modulation of specific signaling pathways. By controlling the enzymes of the Krebs (citric acid) cycle, they contribute to crosstalk between carbon and nitrogen metabolites (Colla et al., 2014; Du Jardin, 2015). The beneficial effect of chelation by some AAs has also been reported. In this way, certain AAs can protect plants from heavy metals, but they also contribute to the mobility and acquisition of micronutrients by the roots. AAs can also reduce environmental stress by scavenging free oxygen radicals, thereby contributing to antioxidant activity (Calvo et al., 2014). The stem and leaves of cannabis, like other plants, contain various concentrations of incorporated AAs (Audu et al., 2014). Plants can absorb and incorporate nitrogen in the form of intact AAs (Persson and Nasholm, 2001; Sauheitl et al., 2009), and thus, solutions of protein hydrolysates and AAs can increase plant growth (El-Ghamry et al., 2009; Talukder et al., 2018) and the nitrogen content of above-ground biomass (Matsumoto et al., 1999). Supplementing plants with environmentally friendly AA biostimulants can reduce the use of inorganic fertilizers (Ugolini et al., 2015).

Several commercial products derived from protein hydrolysates of plant and animal origin have already been marketed. Various results have been reported for agricultural and horticultural crops, but their application has led to significant improvements in yield and quality parameters (Calvo et al., 2014). So far, however, there have been no publications about their effects on plant secondary metabolism. Therefore, in this study, we focused on the physiological and chemical responses of medical cannabis plants to supplementation with a spectrum of AAs in a nutrient solution and subsequently compare the outcomes with two different hydroponic nutritional cycles. We proposed the following hypotheses: (1) the nutritional AA supplement causes a change in the amount of above-ground biomass and affects the inflorescence yield of medical cannabis plants; (2) the nutritional AA supplement causes a change in the medical cannabis plants cannabinoid and terpene profile; (3) the induced changes will be correlated with the contents of macro- and micro-elements in plant organs (leaves, stems, flowers); and (4) the induced changes will differ in each nutrition systems (recirculation vs. drain-to-waste). To test the hypotheses, we monitored the effects of AA supplementation in the nutrient solution of both systems on the amount of above-ground biomass and growth of leaves, stems, and flowers, the concentration of cannabinoids and terpenes, and the tissue ionome of the medical cannabis plant.

## Materials and Methods

### Basic Parameters of the Growing Space

Cannabis plants were grown on tables in a room with controlled conditions. Each 2 m^2^ (1 × 2 m) table supported a separate experiment with an independent 100 l tank for the nutrient solution. The container was made of inert plastic certified for food industry use. Each table held a maximum of 55 black conical square pots made of polypropylene (PP), each with a volume of 3.45 l with dimensions: TOP - 15 cm x 15 cm, BASE - 11.5 cm x 11.5 cm, HEIGHT −20 cm. Irrigation was provided by capillaries, which were placed in each pot to reach every plant separately using a needle applicator. The pump’s timer was set for nine irrigation cycles, each lasting 60 s. During one cycle, 94 ml of nutrient solution was supplied to each plant (846 ml per plant per day). The growing tables allowed us to choose the irrigation method--either recirculation of the nutrient solution or drain-to-waste system, where the spent solution went to a separate waste tank and was no longer mixed with the original solution. Microclimatic parameters were provided by an air ventilation unit that maintained and recorded the set parameters (relative humidity, temperature, CO_2_ level). Enrichment of the atmosphere of the growing space with CO_2_ was made possible by a generator that burned methane. Six double-ended high-pressure sodium lamps provided a suitable spectrum of light at a power of 1,000 W. Based on the photosynthetic photon flux density (PPFD), the lamps provided 1,029 μmol/m^2^ s at a power of 6,000 W. The light mode was also recorded every minute using a data logger.

### Plants and Growing Conditions

The plants used in the experiments came from the mother plants of the medical cannabis genotype with the working name “McLove.” Plants are classified as chemotype I - high Δ^9^-tetrahydrocannabinolic acid/cannabidiolic acid (THCA/CBDA) ratio (> > 1.0). Appropriate mother plants were kept in a separate growing room with controlled conditions. A total of 220 cuttings were made (110 cuttings per cycle) and cultivated for 21 days in a rock-wool cube (4 × 4 cm). Rooted clones were moved to a growing room, where they were placed in 3.45-liter pots filled with three liters of Euro Pebbles (expanded clay) growing medium. The light mode was set to 18 h of light and 6 h of darkness, temperature in the light phase was 25°C, the relative humidity was 60%, and the CO_2_ concentration was 550 ppm (1,065 mg/m^3^). The dark phase temperature was reduced to 22°C with the same humidity. The vegetative phase lasted 7 days, after which the cultivation regime was adjusted to the generative phase. The light period was set at 12 h light and 12 h dark, the temperature and CO_2_ concentration was left the same as the vegetative phase, and the relative humidity was reduced to 40%. From the 10^th^ week, plants were irrigated with demineralized water (DMW). Plant density was 27.5 plants per m^2^ (55 plants/table/treatment).

### Treatments

Compared to the controls (CN), the experimental plants (ET) were exposed to one enhanced nutrition treatment with two separate nutritional cycles. The first cycle (1C) was performed with recirculated nutrient solution, and the second cycle (2C) used the drain-to-waste system. The enhanced treatments were set up for both nutritional cycles and received the AA biostimulant (Table 1) added from the 2nd week for the last 24 h at a volume 2 ml/l before changing the nutrient solution. The new nutrient solution was prepared from reverse osmosis water every 7 days from the first day of the experiment. The pH of the nutrient solution was adjusted to 5.9 (Velazquez et al., 2013). In the recirculation system the nutrient solution was adjusted to this value every day. The pH and electrical conductivity (EC) were recorded when mixing the new solution and on the last day before changing it. After preparing the fresh nutrient solution, a sample was taken from each treatment for analysis. The measured composition of the control treatment (CN) nutrient solution is shown in Table 2, and the composition of the enhanced treatment (ET) nutrient solution with the addition of AAs is shown in Table 3.

**Table 1:** Amino acid content in biostimulant.

AA	mg/L
Lys	0.071
His	0.00483
Arg	0.04615
Asp	0.0327
Thr	0.00954
Ser	0.0175
Glu	0.062
Pro	0.0828
Gly	0.1449
Ala	0.05569
Cys	0.036
Val	0.01401
Met	0.0039
Ile	0.00966
Leu	0.01836
Tyr	0.0016
Phe	0.01305

**Table 2:** Composition of control treatment (CN) nutrient solution (mg/L).

Elements	Weeks				
	1	2	3, 5	4, 6–9	10–13
N	100.85 ± 1.64	116.00 ± 1.85	130.00 ± 1.75	150.00 ± 1.92	DMW^a^
P	32.01 ± 0.75	39.40 ± 0.82	43.88 ± 0.59	51.73 ± 0.79	DMW^a^
K	124.93 ± 1.85	151.00 ± 1.38	173.11 ± 1.92	193.25 ± 1.58	DMW^a^
Ca	98.53 ± 1.32	119.00 ± 1.35	132.38 ± 1.42	146.00 ± 1.28	DMW^a^
Mg	25.17 ± 0.38	30.50 ± 0.42	34.94 ± 0.48	39.13 ± 0.45	DMW^a^
S	21.75 ± 0.25	26.72 ± 0.29	31.34 ± 0.34	34.53 ± 0.38	DMW^a^
Fe	0.91 ± 0.09	1.11 ± 0.09	1.21 ± 0.11	1.44 ± 0.08	DMW^a^
Mn	0.66 ± 0.07	0.74 ± 0.05	0.83 ± 0.08	0.99 ± 0.07	DMW^a^
Zn	0.21 ± 0.03	0.27 ± 0.03	0.28 ± 0.04	0.33 ± 0.03	DMW^a^
Cu	0.07 ± 0.01	0.09 ± 0.01	0.11 ± 0.01	0.13 ± 0.02	DMW^a^
B	0.14 ± 0.02	0.19 ± 0.01	0.22 ± 0.02	0.25 ± 0.02	DMW^a^
Mo	0.01 ± 0.00	0.02 ± 0.00	0.02 ± 0.00	0.02 ± 0.00	DMW^a^
EC	0.97 ± 0.01	1.19 ± 0.01	1.46 ± 0.01	1.74 ± 0.01	DMW^a^

a *demineralized water.*

**Table 3:** Composition of enhanced treatment (ET) nutrient solution with the addition of AAs (mg/L).

Elements	Weeks				
	1	2	3, 5	4, 6–9	10–13
N	100.00 ± 1.59	300.00 ± 2.94	331.00 ± 3.01	353.00 ± 3.52	DMW^a^
P	32.20 ± 0.49	40.17 ± 0.52	44.18 ± 0.92	52.09 ± 0.57	DMW^a^
K	125.00 ± 1.56	151.51 ± 1.27	174.17 ± 1.38	194.26 ± 1.95	DMW^a^
Ca	98.50 ± 1.32	120.58 ± 1.24	133.15 ± 1.49	146.83 ± 1.56	DMW^a^
Mg	25.30 ± 0.34	31.00 ± 0.38	34.06 ± 0.43	40.03 ± 0.37	DMW^a^
S	21.49 ± 0.31	51.80 ± 0.52	56.27 ± 0.61	61.84 ± 0.85	DMW^a^
Fe	0.93 ± 0.08	1.14 ± 0.07	1.19 ± 0.09	1.47 ± 0.07	DMW^a^
Mn	0.64 ± 0.06	0.75 ± 0.03	0.81 ± 0.04	1.01 ± 0.07	DMW^a^
Zn	0.22 ± 0.04	0.27 ± 0.01	0.29 ± 0.02	0.36 ± 0.03	DMW^a^
Cu	0.07 ± 0.01	0.09 ± 0.02	0.11 ± 0.02	0.13 ± 0.01	DMW^a^
B	0.15 ± 0.01	0.20 ± 0.02	0.22 ± 0.02	0.26 ± 0.01	DMW^a^
Mo	0.01 ± 0.00	0.02 ± 0.00	0.02 ± 0.00	0.03 ± 0.00	DMW^a^
EC	0.97 ± 0.01	1.38 ± 0.01	1.71 ± 0.01	2.14 ± 0.01	DMW^a^

a *demineralized water.*

### Sampling Plant Material

**Figure 1:**
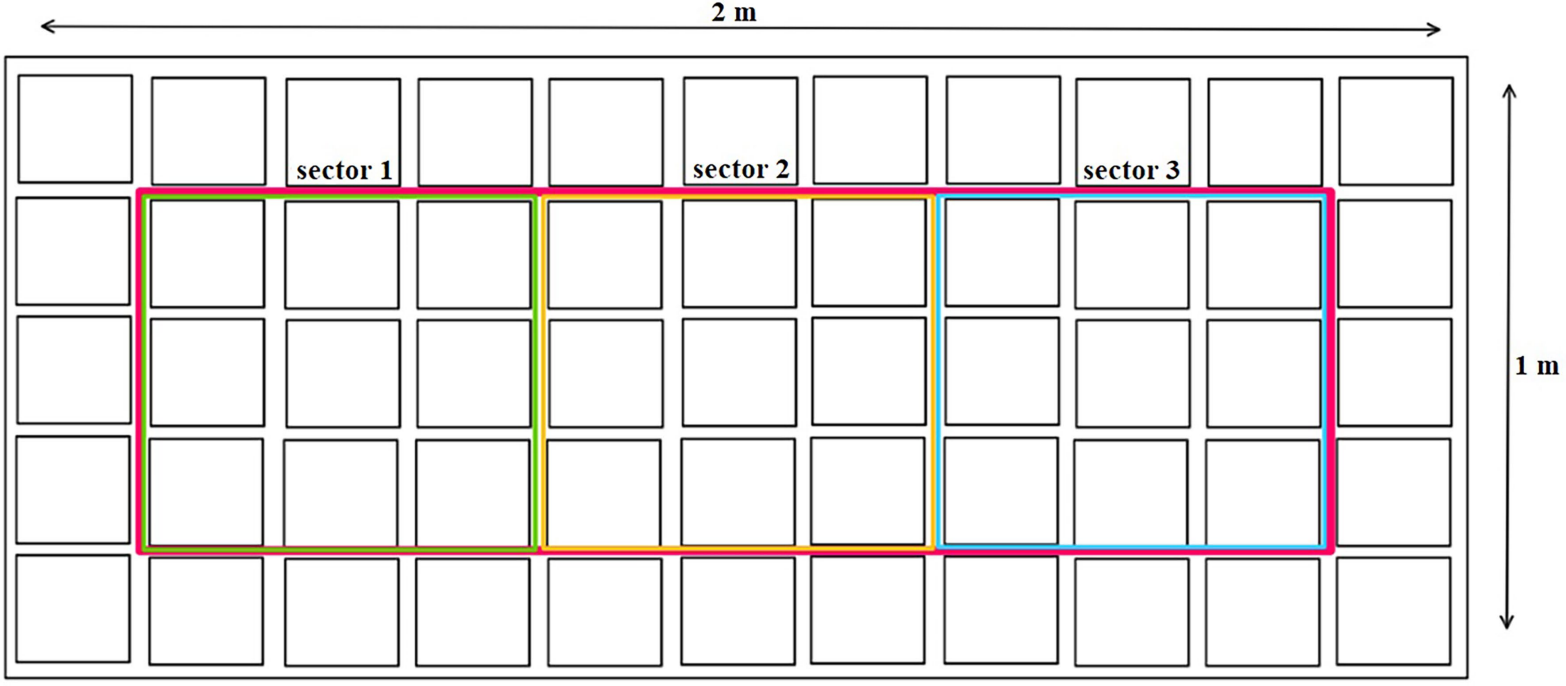
Plant sampling method.

Three plants were harvested from each treatment, one plant randomly selected from each highlighted sector 1–3 (Figure 1), every 7 days during the entire vegetation cycle. Subsequently, a random plant from the edge (outside the sectors) was transferred to an empty space in each sector. Plants were uprooted, weighed whole fresh, and divided into leaves, stems, and flowers, which were weighed fresh separately for all plants. The materials were then dried at 25°C to constant moisture (8–10%) and re-weighed. To determine the dry matter, a reference amount of each part of the plant was dried at 105°C to constant weight. The plant parts were homogenized just before analysis. The flowers (including the leaves until the 4th week) were frozen in liquid nitrogen and then ground in a mortar and pestle. The dried leaves (from the 5th week) and stems were ground in a grinder.

### Dry Decomposition and Elemental Analysis

To determine the content of macroelements (except nitrogen), microelements, and trace elements in the plant, the leaves, stems, and flowers were analyzed separately. Weighed and homogenized plant biomass in a beaker was covered with a watch glass, placed on a hotplate 160°C, and the temperature was raised to 350°C over 4 h during which the samples gradually decomposed. The samples were next transferred to a muffle furnace, where they remained at 450–500°C for 12 h (Miholová et al., 1993). One ml of 65% HNO_3_ was then added to the cooled beakers, which were placed on a 120°C hot plate for 60 min. The samples were then annealed for 90 min in an oven at 500°C and suspended in 1.5% HNO_3_ with stirring in an ultrasonic bath. Elemental analysis of the samples was performed by flame atomic absorption spectrometry (FAAS) on a Varian 280FS with inductively coupled plasma optical emission spectrometry (ICP-OES) by Varian Vista-PRO instrument (Varian, Mulgrave, Australia; Hoenig, 2003).

### Determination of Nitrogen in Plant Material by the Kjeldahl Method

For nitrogen determination, 0.5 g of plant material was weighed and put into a distillation tube. The samples were then mineralized by boiling with 95% H_2_SO_4_. After alkalization with sodium hydroxide, the free ammonia was distilled with water steam into H_3_BO_3_. Its content was determined by titration with HCl (0.5 mol/l) and then measured by Gerhardt Vapodest 30s (Königswinter, Germany; Baker and Thompson, 1992; Velechovský et al., 2021).

### Phytocannabinoid Extraction, Identification, and Quantification

Phytocannabinoids from ground homogenized flowers (including the leaves until the 4th week) were extracted by the optimized method of dynamic maceration (Brighenti et al., 2017). Samples (0.30 g) from each experiment group were mixed with 10 ml of 96% ethanol and macerated for 60 min with constant stirring at 300 rpm. Mixtures were then filtered under vacuum using a Morton filter device (porosity S4/P16), and the filtrate was collected. The flowers were removed from the filter and mixed with another 10 ml of solvent. This step was repeated twice, and the filtrates were pooled. Aliquots of 0.5 ml of each sample were diluted to 10 ml with 96% ethanol and filtered once more through nylon syringe filters (0.22 μm) into vials. Samples of the extracts were injected into high-performance liquid chromatography system equipped with diode array detection (HPLC-DAD; Agilent 1,260, Agilent Technologies Inc., United States) and a Luna^®^ C18 column (2) 250 × 3 mm, particle size 3 μm (Phenomenex, United States). The isocratic mobile phase consisted of acetonitrile/H_2_O (31:9, v/v) with 0.1% HCOOH (v/v) and 0.1 mol/l NH_4_COOH (without pH adjustment), flow rate was 0.55 ml/min, temperature 37°C, sample injection volume 8 μl, and UV detection at 275 nm (Križman, 2020). The instrument was externally calibrated using cannabinolic acid (CBNA) in the range of 0.3–10 mg/l and THCA, 0.3–100 mg/l, (Sigma-Aldrich, Czech Republic) as standards. Data were analyzed using OpenLAB CDS software, ChemStation Edition, Rev. C.01.5.

### Terpene Extraction, Identification, and Quantification

Terpenes from ground and homogenized mature flowers (week 8–10, vegetation) were extracted with hexane. Plant samples (0.1 g) were mixed with 1 ml of hexane and pentadecane was added to a final concentration of 1 mg/ml as an internal standard. The samples were vortexed and placed in an ultrasonic bath for 30 min. Subsequently, the samples were centrifuged and filtered through polytetrafluoroethylene (PTFE) syringe filters (0.22 μm) into vials. Filtered samples (1.5 μl) were first injected into a gas chromatograph with a flame-ionization detector (GC-FID; Agilent Technologies 7890A, Palo Alto, CA). The GC-FID conditions were: column DB5 30 m × 0.25 mm × 0.25 μm film thickness, inlet temperature 230°C, detector temperature 300°C, and nitrogen flow rate of 1 ml/min. The initial temperature was 60°C, which was increased at the rate of 3.5°C/min until a temperature of 150°C was reached, and then at a rate of 30°C/min until a final temperature of 300°C was reached. Samples were also injected into a gas chromatograph connected to a mass spectrometer (GC–MS; Agilent Technologies 5975C, Palo Alto, CA). The GC–MS conditions were: column HP-5MS 30 m × 0.25 mm × 0.25 μm film thickness, inlet temperature 230°C, detector temperature 300°C, and helium flow rate of 1 ml/min. The initial temperature was 60°C, which was increased at the rate of 3.5°C/min until a temperature of 150°C was reached, and then at a rate of 30°C/min until a final temperature of 300°C was reached. Compounds detected by GC–MS were identified by comparing the mass spectrum and relative retention index with the published values of the National Institute of Standards and Technology (NIST) database, and the values for the standards, β-myrcene and limonene (Sigma-Aldrich, Czech Republic). The GC-FID data revealed the relative concentration of the identified substances, based on the peak area of the monitored substance relative to the total area of all detected substances.

### Statistical Analyses

Data were subjected to ANOVA followed by Tukey’s HSD test. The analysis was performed using IBM SPSS Statistics software (version 25, 2017, Chicago, Illinois, United States).

## Results

**Figure 2:**
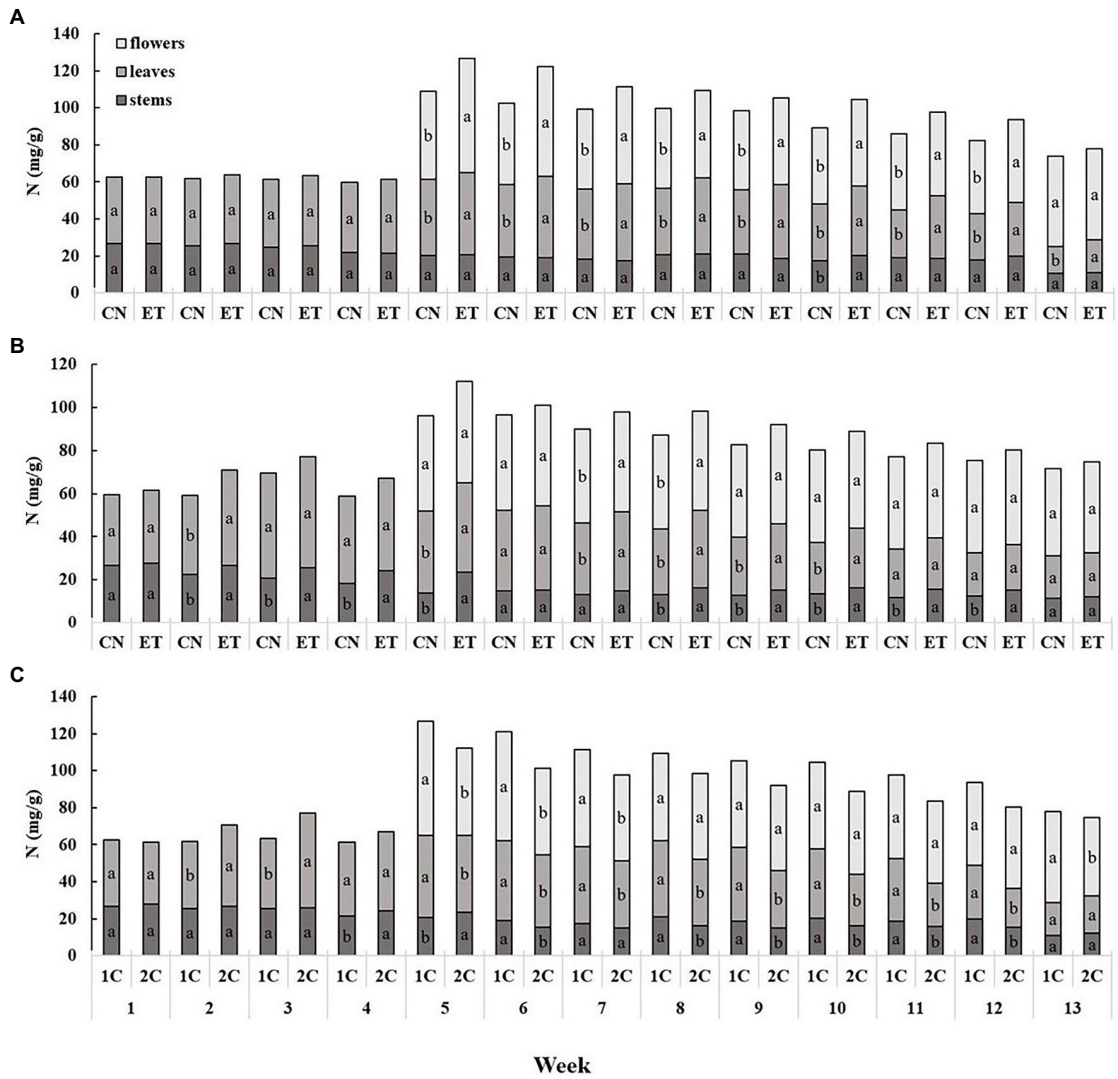
Distribution of nitrogen among the organs of medical cannabis plants as affected by amino acid (AA) supplementation and nutrient cycle. N concentration of control (CN) and enhanced treatment (ET) with AAs nutritional supplement in recirculation (1C) growing cycle **(A)**, in drain-to-waste (2C) growing cycle **(B)**, ETs in 1C and 2C **(C)** in stems, leaves, and flowers. Data are means ± SE (*n* = 3). The different small letters inside the bars represent significant differences within the plant organs (stems, leaves, and flowers) between the variants in a particular week according to Tukey’s HSD test at *α* = 0.05.

The AA nutritional supplement and the variable nutritional cycles (1C and 2C) induced changes in the tissue ionome of medical cannabis plants. The content of nitrogenous compounds was lowest in the stems and highest in the flowers (Figure 2). The concentrations of N in the leaves and flowers of control (CN) and enhanced treatment (ET) plants with AA supplement in the recirculation (1C) cycle began to differ significantly from the 5th week. The most significant differences in N concentrations between control and AA treatment were 34% for flowers at week 6 (CN, 44.26 mg/g; ET, 59.19 mg/g; Figure 2A). In contrast to 1C, the concentration of N in the stems and leaves of CN and ET began to differ significantly from week 2 to 4 in the drain-to-waste (2C) nutritional cycle; but, from week 5 to 13, fewer significant differences were observed with 2C than 1C. The most significant differences in N concentrations between nutritional treatments were 7% for flowers at week 7 (CN, 43.02 mg/g; ET, 45.85 mg/g; Figure 2B). The N concentration also differed between 1C and 2C of ETs with AA supplement, and the differences were evident beginning at week 2. The most significant differences in N concentrations in ETs between nutritional cycles were 31% (6% between CNs) for flowers at week 5 (1C, 61.63 mg/g; 2C, 47.18 mg/g; Figure 2C).

**Figure 3:**
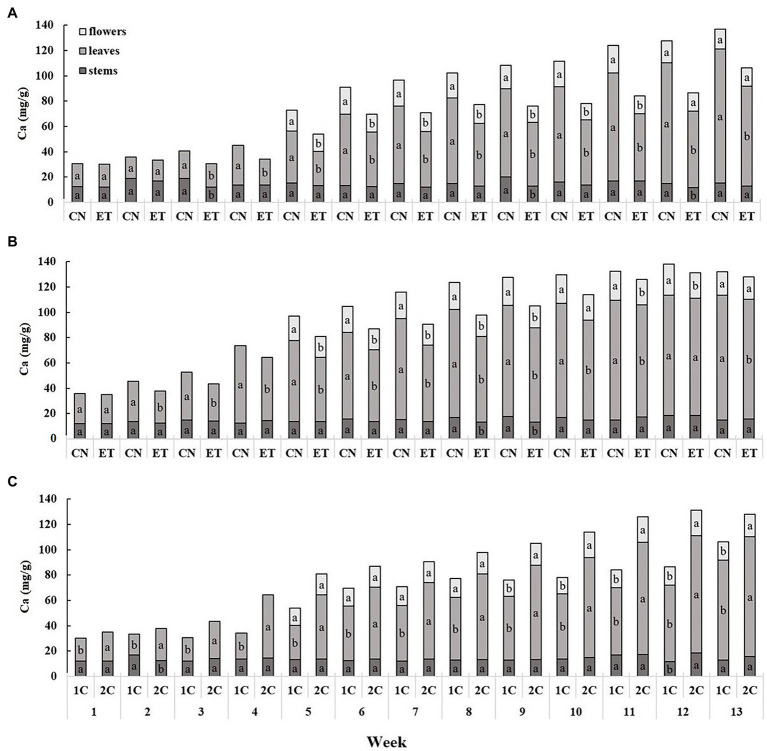
Distribution of calcium among the organs of medical cannabis plants as affected by amino acid (AA) supplementation and nutrient cycle. Ca concentration in control (CN) and enhanced treatment (ET) plants with AAs nutritional supplement in recirculation (1C) growing cycle **(A)**, in drain-to-waste (2C) growing cycle **(B)**, ETs in 1C and 2C **(C)** in stems, leaves, and flowers. Data are means ± SE (*n* = 3). The different small letters inside the bars represent significant differences within the plant organs (stems, leaves, and flowers) between the variants in a particular week according to Tukey’s HSD test at *α* = 0.05.

The calcium content was lowest in the stems and highest in the leaves, and showed a cumulative trend over time (Figure 3). The Ca concentration in the leaves of CN and ET in 1C began to differ significantly from the third week. The most significant differences in Ca concentration between nutritional treatments were 60% for leaves at week 11 (CN, 85.17 mg/g; ET, 53.13 mg/g; Figure 3A). In contrast to 1C, the Ca concentration in the CN and ET leaves differed significantly as early as week 2 in 2C. The most significant differences in Ca concentrations between CN and ET were 32% for leaves at week 7 (CN, 79.60 mg/g; ET, 60.13 mg/g; Figure 3B). The Ca concentration in ET also varied between 1C and 2C, beginning at week 1. The most significant differences in Ca concentrations in ETs between 1C and 2C in the last weeks of vegetation growth were 67% (11% between CNs) for leaves at week 11 (1C, 53.13 mg/g; 2C, 88.87 mg/g; Figure 3C).

**Figure 4:**
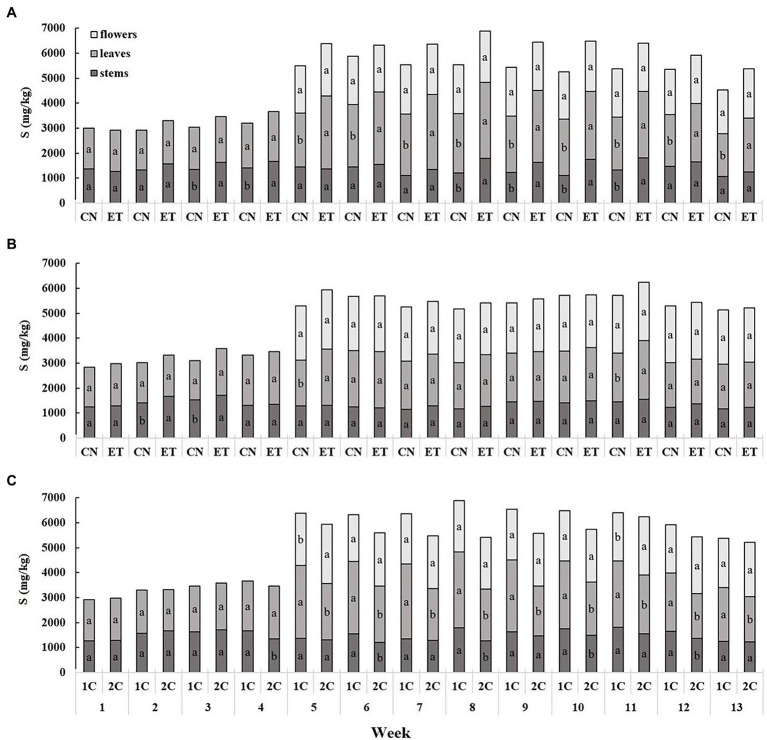
Distribution of sulfur among the organs of medical cannabis plants as affected by amino acid (AA) supplementation and nutrient cycle. S concentration in control (CN) and enhanced treatment (ET) plants with AAs nutritional supplement in recirculation (1C) growing cycle **(A)**, in drain-to-waste (2C) growing cycle **(B)**, ETs in 1C and 2C **(C)** in stems, leaves, and flowers. Data are means ± SE (*n* = 3). The different small letters inside the bars represent significant differences within the medical cannabis plant organs (stems, leaves, and flowers) between the variants in a particular week according to Tukey’s HSD test at *α* = 0.05.

The content of sulfur compounds was the lowest in the stems and the highest in the leaves (Figure 4). The concentration of S in the stems and leaves for CN and ET with 1C began to differ significantly from the third week. The most significant differences in S between CN and ET were 28% for leaves in week 8 (CN, 2375 mg/kg; ET, 3029 mg/kg; Figure 4A). In contrast to 1C, the concentration of S in the stems and leaves of CN and ET began to differ significantly from the second week for 2C; however, fewer significant differences than in 1C were observed. The most significant differences in S concentrations between nutritional treatments were 23% for leaves at week 5 (CN, 1834 mg/kg; ET - 2,260 mg/kg; Figure 4B). The S concentration also varied between 1C and 2C of ETs but was almost identical till the 3^rd^ week. The most significant differences in S concentrations in ETs between 1C and 2C were 46% (27% between CNs) for leaves at week 8 (1C, 3,029 mg/kg; 2C, 2068 mg/kg; Figure 4C).

**Figure 5:**
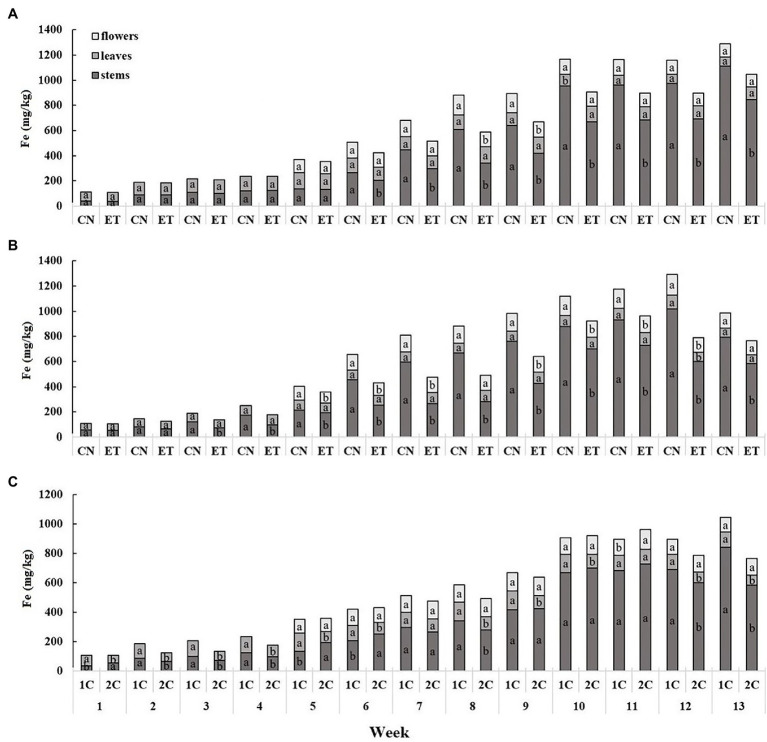
Distribution of iron among the organs of medical cannabis plants as affected by amino acid (AA) supplementation and nutrient cycle. Fe concentration in control (CN) and enhanced treatment (ET) plants with AAs nutritional supplements in recirculation (1C) growing cycle **(A)**, in drain-to-waste (2C) growing cycle **(B)**, ETs in 1C and 2C **(C)** in stems, leaves, and flowers. Data are means ± SE (*n* = 3). The different small letters inside the bars represent significant differences within the medical cannabis plant organs (stems, leaves, and flowers) between the variants in a particular week according to Tukey’s HSD test at *α* = 0.05.

The iron content was the lowest in leaves and highest in stems and showed a cumulative trend over time (Figure 5). The concentration of Fe in the stems for CN and ET in 1C began to differ significantly from week 6 to 13. The most significant differences in Fe between CN and ET were 79% for stems at week 8 (CN, 609.5 mg/kg; ET, 340.6 mg/kg; Figure 5A). In contrast to 1C, the concentration of Fe in the stems of the CN and ET began to differ significantly from week 3 to 13 in 2C. The most significant difference in Fe concentrations between CN and ET was 139% for stems at week 8 (CN, 666.4 mg/kg; ET, 279.3 mg/kg; Figure 5B). The Fe concentration also varied between 1C and 2C of ETs by the first week. The most significant difference in Fe concentrations in ETs between 1C and 2C in the last weeks of vegetation growth was 45% (40% between CNs) for stems at week 13 (1C, 844.2 mg/kg; 2C, 584.4 mg/kg; Figure 5C).

**Figure 6:**
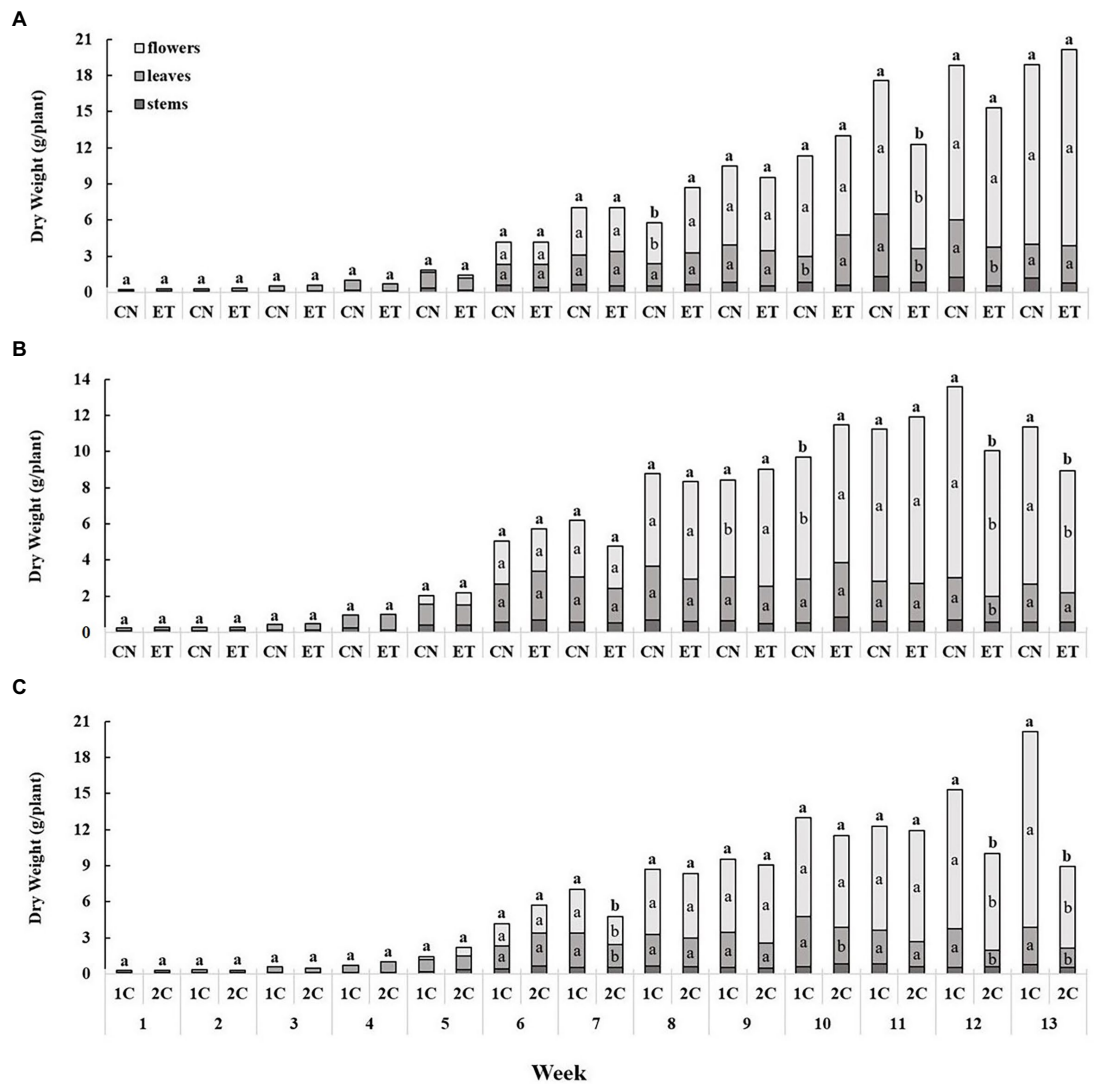
The effect of amino acid supplementation (AAs) and growing nutritional cycle on medical cannabis plant biomass. Dry biomasses of stems, leaves, and flowers in control (CN) and enhanced treatment (ET) plants with AAs nutritional supplements in recirculation (1C) growing cycle **(A)**, in drain-to-waste (2C) growing cycle **(B)**, ETs in 1C and 2C **(C)**. Data are means ± SE (*n* = 3). The different small letters inside the bars and small bold letters above the bars represent significant differences within the medical cannabis plant organs (leaves and flowers) and the whole plant biomass between the variants in a particular week according to Tukey’s HSD test at *α* = 0.05.

Nutritional supplementation with AAs in the two different nutritional cycles caused some change in growth of medical cannabis plants. Up to week 5, the increase in biomass was relatively slow, but was sharply increased from week 6. The largest weekly dry weight gain was recorded for flowers (Figure 6). The increase in biomass of stems, leaves, and flowers for CNs and ETs in 1C was almost identical until week 7. From week 8 to 12, leaf and flower biomass differed somewhat (Figure 6A). In contrast to 1C, stems, leaves, and flowers of CN and ET plants in 2C increased significantly from week 9. ET reached maximum dry plant biomass at week 11, and CN by week 12 (Figure 6B). Biomass also varied between 1C and 2C of ETs, but from week 7 (Figure 6C).

**Figure 7:**
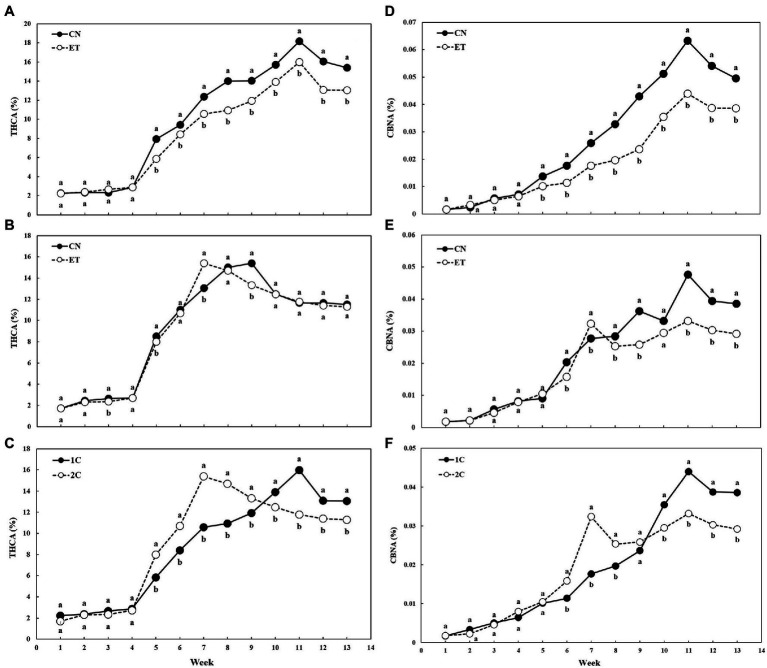
Concentrations of tetrahydrocannabinolic acid (THCA) and cannabinolic acid (CBNA) in the flowers of medical cannabis plants as affected by amino acid (AA) supplementation and nutrient cycle. THCA concentration in control (CN) and enhanced treatment (ET) plants with AAs nutritional supplements in recirculation (1C) growing cycle **(A)**, in drain-to-waste (2C) growing cycle **(B)**, ETs in 1C and 2C **(C)**. CBNA concentration of control (CN) and enhanced treatment (ET) with AAs nutritional supplements in recirculation (1C) growing cycle **(D)**, in drain-to-waste (2C) growing cycle **(E)**, ETs in 1C and 2C **(F)**. The whole inflorescence of the plant was analyzed. Data are means ± SE (*n* = 3). Different bold small letters represent significant differences in cannabinoid concentration between the variants in a particular week according to Tukey’s HSD test at *α* = 0.05.

AA supplementation and nutritional cycle changed the concentration of THCA and CBNA in the flowers of cannabis plants, but concentration curves of both cannabinoid acids were similar for the same nutritional cycle and treatment (Figure 7). THCA in leaves and flowers slowly increased in both treatments until week 4, but from week 5, the concentration of THCA began to differ significantly because only flowers were analyzed (Figures 7A–C). The CN and ET concentrations of THCA began to differ significantly from week 5 to 13 in 1C, and CN (18.2%) and ET (16.0%) reached maximum at week 11 (Figure 7A). In contrast to 1C, the concentration of THCA in CN and ET (2C) differed significantly by the third week, but the differences were smaller. THCA peaked at week 9 for CN (15.4%) and week 7 for ET (15.4%; Figure 7B). The THCA levels in 1C and 2C of ETs differed significantly from week 5–13 (Figure 7C). The CBNA concentration in CN and ET in 1C began to vary significantly between week 5 and 13. CBNA peaked at week 11 in both treatments and differed significantly by 44% (Figure 7D). In contrast to 1C, the CBNA concentration in CN and ET did not differ significantly in 2C until weeks 5 and 10. CBNA in CN reached two maxima in 2C: at week 9, where it differed significantly by 41%, and at week 11 where it differed significantly by 44%. The CBNA for ET also reached two maxima in 2C: at week 7 where it differed significantly by 17%, and at week 11, the same as CN (Figure 7E). CBNA concentrations between 1C and 2C of ETs were almost identical until weeks 5 and 9. As stated above, the CBNA concentration of ET reached maximum at week 11 in 1C, when it differed significantly by 33% (also 33% for CNs) and at week 7 in 2C, when it differed significantly by 83% (7% for CNs; Figure 7F).

**Figure 8:**
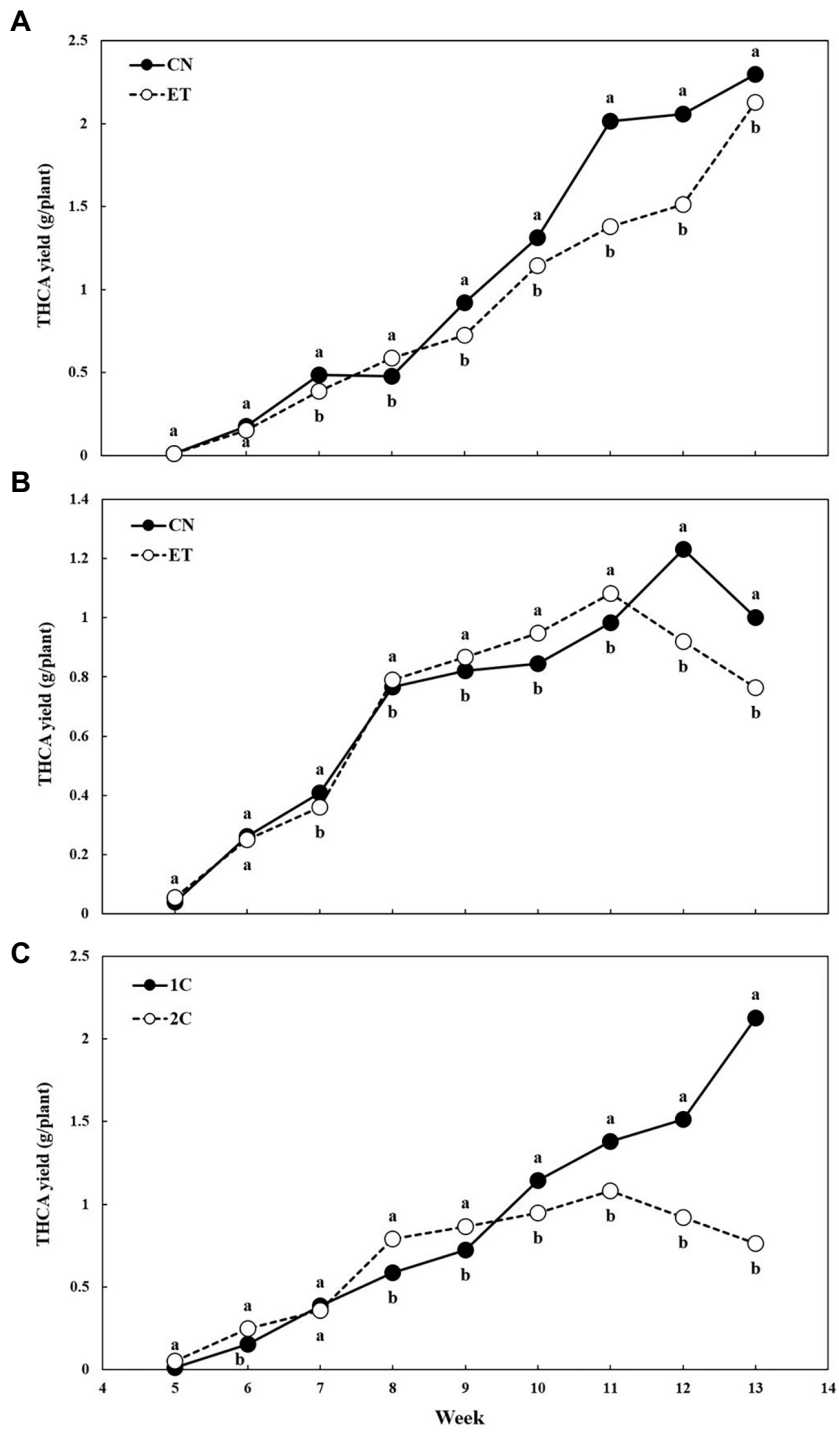
Tetrahydrocannabinolic acid (THCA) yield per plant as affected by amino acid (AA) supplementation and nutrient cycle. THCA yield per plant in control (CN) and enhanced treatment (ET) with AAs nutritional supplements in recirculation (1C) nutrient cycle **(A)**, in drain-to-waste (2C) nutrient cycle **(B)**, ETs in 1C and 2C **(C)**. The whole inflorescence of the plant was analyzed. Data are means ± SE (*n* = 3). Different bold small letters represent significant differences in cannabinoid concentration between the variants in a particular week according to Tukey’s HSD test at *α* = 0.05.

THCA is the most concentrated cannabinoid in our medical cannabis plant chemotype. The THCA yield per plant from dried flowers over time and the effect of the AA supplement and variable nutritional cycle was measured (Figure 8). THCA yields were almost identical for CN and ET with 1C until week 6 but differed significantly from week 7–13. The largest significant difference (46%) between the nutritional treatments was achieved at week 11, but the highest yield for both treatments was at week 13 (Figure 8A). The THCA yield for CN with 2C compared to 1C reached its maximum at week 12 (significant difference, 34%) and for ET at week 11 (significant difference, 10%; Figure 8B). As stated above, the THCA yield for ET with 1C reached a maximum at week 13 (significant difference between ETs, 279%) and at week 11 for ET with 2C (difference between ETs, 28%; Figure 8C).

**Figure 9:**
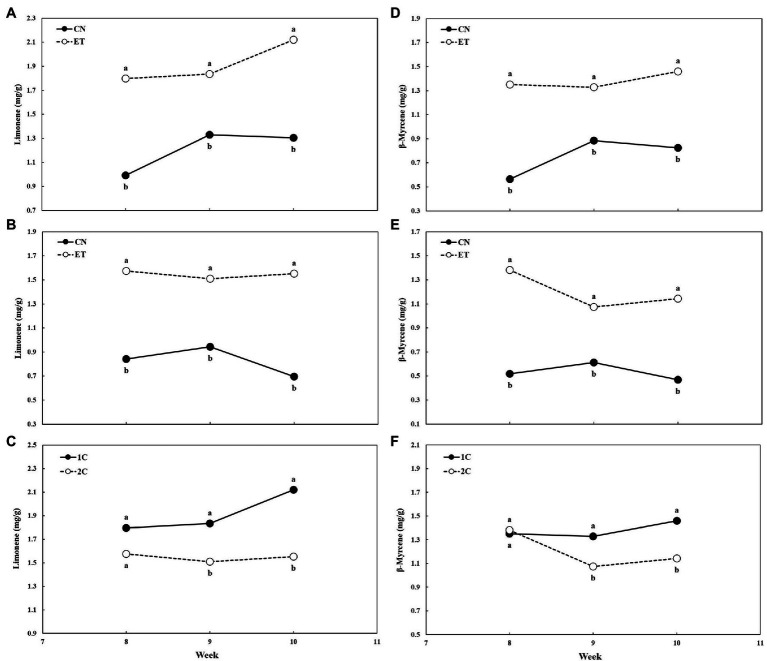
Concentration of limonene and β-myrcene in flowers of medical cannabis plants as affected by amino acid (AA) supplementation and nutrient cycle. Limonene concentration in control (CN) and enhanced treatment (ET) plants with AAs nutritional supplements in recirculation (1C) nutrient cycle **(A)**, in drain-to-waste (2C) nutrient cycle **(B)**, ETs in 1C and 2C **(C)**, the β-myrcene concentration of control (CN) and enhanced treatment (ET) with AAs nutritional supplements in recirculation (1C) growing cycle **(D)**, in drain-to-waste (2C) growing cycle **(E)**, ETs in 1C and 2C **(F)**. The whole inflorescence of the plant was analyzed. Data are means ± SE (*n* = 3). Different bold small letters represent significant differences in cannabinoid concentration between the variants in a particular week according to Tukey’s HSD test at *α* = 0.05.

The concentrations of limonene and β-myrcene in the flowers were also affected by AA supplementation and nutrient cycle (Figure 9). Limonene peaked at week 9 for CN (1.33 mg/g) and at week 10 for ET (2.12 mg/g). The most significant difference in limonene concentration between these two treatments was 81% reached at week 8 in 1C (Figure 9A). As in 1C, limonene concentration peaked at week 9 for CN (0.94 mg/g) but at week 8 for ET (1.58 mg/g) in 2C. The largest significant difference in limonene concentration between these two treatments was 123% reached at week 10 (Figure 9B). Comparing limonene concentrations of ETs for 1C and 2C, the largest significant difference between these two cycles was 37% at week 10 (Figure 9C). β-myrcene levels peaked at week 9 for CN (0.89 mg/g) and at week 10 for ET (1.46 mg/g). The largest significant difference in β-myrcene concentration between these two treatments was 139% at week 8 in 1C (Figure 9D). As in 1C, β-myrcene peaked at week 9 for CN (0.61 mg/g), but at week 8 for ET (1.38 mg/g) in 2C. The largest significant difference in β-myrcene concentration between these two treatments was 167% at week 8 (Figure 9E). Comparing β-myrcene concentration in ETs for 1C and 2C, the most significant difference between these two cycles was 28% reached at week 10 (Figure 9F).

## Discussion

Nutrition is undoubtedly an important factor in the development, function, and metabolism of all plant organs and tissues. Data are already known regarding the optimal levels of individual macronutrients, such as N, P, and K, for normal function and development of the root system and above-ground biomass (Saloner et al., 2019; Saloner and Bernstein, 2020; Shiponi and Bernstein, 2021b) and formation of the desirable secondary metabolites of medical cannabis plants (Caplan et al., 2017a; Bernstein et al., 2019; Saloner and Bernstein, 2021; Yep et al., 2021; Shiponi and Bernstein, 2021a). However, there is still emphasis on the availability of sufficient quantities of these major plant macronutrients in an optimal ratio. The effects of micronutrients (Yep et al., 2021) and plant biostimulants (Bernstein et al., 2019) must also be considered.

Nutritional treatment with AA supplements in different nutrient cycles clearly affected the concentrations of macro- and micro-elements in cannabis plants. Antagonistic and synergistic interactions between nutrient anions and cations during root cell membrane transport have been relatively well reported. However, the timing of replenishment of AAs and variations in pH, as in the case of the recirculation cycle, 1C, could affect their accessibility from the nutrient solution and thus the subsequent physiological and metabolic response of plants. The enhanced treatment (ET) with AA supplementation resulted in significantly greater nitrogen accumulation (Figures 2A,B) in all three plant organs, but mostly in flowers and leaves. This finding is consistent with claims that plants can absorb and incorporate intact amino acids directly (Matsumoto et al., 1999; Persson and Nasholm, 2001; Jämtgård et al., 2008). AAs can also modulate the assimilation and absorption of N in plants by regulating the enzymes and structural proteins involved in these processes. AAs also affect N uptake signaling pathways in roots and promote transfer between nitrogen and carbon metabolites by controlling enzymes of the tricarboxylic acid cycle (Colla et al., 2014; Du Jardin, 2015). When comparing nutritional cycles (Figure 2C), higher N concentrations were observed in the above-ground organs of plants, especially in leaves and flowers from ET plants in 1C. This was probably due to fluctuations in the pH of the 1C nutrient solution from addition of AAs, which increased the pH to 8.05 after 24 h. The initial pH of the nutrient solution, 5.9 (the constant pH of the 2C nutrient solution), was close to the isoelectric point of most AAs (Pogliani, 1992), but recirculation may have resulted in the formation of a partial charge on some AA molecules. At pH 5.9, most AAs were in the neutral zwitterionic form, making them less able to enter plant cells because of lipophilic interactions during membrane transport (Trapp, 2004). Sulfur showed an accumulation trend similar to N, but at a lower concentration (Figure 4), because of the sulfur-containing AAs, cysteine and methionine (Table 1). In 2C (Figure 4B), the S concentrations were almost identical in both treatments, probably because of lower solubility of the sulfur AAs at pH 5.9 and reduced absorption.

Calcium accumulation followed an opposite trend (Figure 3). In the ET group, the AA supplementation significantly lowered calcium accumulation (Figures 3A,B) in all three plant organs, but mostly in leaves and flowers. The same trend was observed for magnesium accumulation (*data not shown*), but with minor differences because of lower concentration. This was probably due to the coordination of calcium with the carboxyl, hydroxyl, thiol, and amino groups of the AAs to form complexes with limited accessibility (Maeda et al., 1990). When comparing nutritional cycles (Figure 3C), higher calcium concentrations were observed in above-ground parts, especially leaves and flowers, from ET plants in 2C. This was probably due to the stable 5.9 pH of the 2C nutrient solution, in which the AAs were in the form of zwitterions that did not complex with *Ca.* The increased formation of root exudates containing negatively charged or free electron pair groups capable of coordinating and binding Ca from the nutrient solution might also have contributed to this process. It is probable that more exudates were excreted in 1C because of the pH change in the cytosol and also from the increase in TCA cycle function after uptake of negatively charged AAs (Ryan et al., 2001). In the case of 2C, replenishment with fresh nutrient solution also contributed to increased calcium ions. Iron showed an accumulation trend similar to calcium, only at lower concentrations, where it occurred mainly in the stem due to low mobility (Figure 5). However, when comparing nutritional cycles (Figure 5C), a higher Fe concentration was observed at some weeks in above-ground organs, especially leaves and stems, of ET plants in 1C. This may have resulted from the Fe levels of ET plants in 2C reaching a maximum at week 11 compared to week 13 in 1C, and also, from the chelating effects of some AAs, which could contribute to mobility and micronutrient acquisition by roots (Calvo et al., 2014). The levels of phosphorus and potassium (*data not shown*) did not differ in nutrient solutions, nor did they show many significant differences in accumulation in the above-ground organs of both treatments, so they were not discussed.

The changed accessibility and supply of individual nutrients within CN and ET plants during different nutritional cycles also affected the yield of dry biomass of stems, leaves, and flowers (Figure 6). In CN with 1C, only a slight increase in the weight of above-ground biomass was observed from week 11 to 13, whereas in ET we saw a sharp increase in total dry matter, especially in flowers, in the last weeks (Figure 6A). This was probably caused by an increased supply of nitrogenous and possibly other compounds in the root cells of ET plants and their subsequent transport to flowers during the so-called rinsing period (watering only with DMW; Table 3) from week 10–13 (Pratelli and Pilot, 2014; Yao et al., 2020). In CN plants with 2C, the maximum increase in biomass was reached at week 12, and in ET a week earlier (Figure 6B). This probably resulted from earlier maturation of the plants with 2C compared to 1C. The differences in dry biomass in the CN and ET groups in both cycles were mainly due to the different N doses from AAs delivered to ET plants from the second (first blooming) week (Tables 2 and 3). According to Saloner and Bernstein (2021), the optimal dose of mineral N for medical cannabis in bloom is 160 mg/l. In our experiments, the amount of mineral N in the nutrient solution was gradually increased from 116 mg/l (week 2) to 150 mg/l (weeks 4 and 6–9) in both CN and ET. Caplan et al. (2017a) stated that the optimal dose of N in organic fertilizers for maximum biomass of medical cannabis plants in bloom was 283 mg/l. In our experiments, the amount of organic N in the nutrient solution for ET plants was gradually increased from 184 mg/l (week 2) to 203 mg/l (weeks 4 and 6–9). However, the amount of total N supplied in the nutrient solution for ET ranged from 300 mg/l (week 2) to 353 mg/l (weeks 4 and 6–9; Table 3). Therefore, this amount of total nitrogen in the nutrient solution may already have exceeded the optimal dosage for medical cannabis plants, especially with 2C (Albornoz, 2016).

This hypothesis was partially supported by the premature ripening of plants based on the concentration of THCA in ET in 2C (Figure 7B), but this could also be caused by increased abiotic stress from high N doses (Gepstein and Glick, 2013). Conversely, the higher dose of nutrients in 2C compared to 1C ensured optimal fertigation, which can shorten the ripening time of cannabis (Caplan et al., 2017b). This hypothesis was supported by the nearly identical trend of increasing THCA concentration with 2C in both treatments, although ET peaked at week 7 compared to CN at week 9 (Figure 7B). When comparing ET results at 1C and 2C, the difference was 4 weeks because the ET plants with 1C did not reach their maximum THCA concentrations until the 11th week (Figure 7C). Differences in THCA concentrations in both treatments and cycles, but especially in 1C, could be explained by the previously discovered positive correlation of calcium with Δ^9^-tetrahydrocannabinol (Δ^9^-THC), which is a decarboxylation product of THCA (Figures 3, 7; Pate, 1994). Its oxidation product, CBNA, had a similar course and maxima as THCA, but reversed (Figures 7D–F), probably because of the antioxidant activity of AAs, which reduced environmental stress by scavenging free oxygen radicals (Calvo et al., 2014).

The combination of the dry weight of flowers and the concentration of THCA was reflected in the yield of THCA. In 1C, an almost linear dependence of THCA yield on time could be seen for both treatments (Figure 8A) because of the lower amount of total nutrients supplied in 1C compared to 2C, and thus the delay in ripening time. However, when comparing ETs from both cycles at the weeks of their maximum THCA yield (week 13 for 1C and week 11for 2C), the THCA yield with 1C was more than twice as high (Figure 8C). This may have been a result of the increased production of abscisic acid (ABA) in response to stress, which slows plant growth and increases THCA production (Mansouri et al., 2009). It was also likely to cause oxidative stress (Jiang and Zhang, 2001), thus indirectly increasing CBNA production (Figure 7F).

The final concentration of monoterpenes showed the same trend in the respective weeks in both cycles and treatments as the concentration of THCA (Figure 9). This was consistent with Aizpurua-Olaizola et al. (2016) who claimed that this could be explained by the fact that monoterpenes were synthesized in the same glandular trichomes as cannabinoids (Meier and Mediavilla, 1998). Similar to Saloner and Bernstein (2021), our results showed that the increased N in the nutrient solution decreased THCA concentration proportionally. But conversely when exceeding a specific limit of nitrogen fertilization, as 160 mg N/L in the case of Saloner and Bernstein (2021), a reversible increase in limonene and myrcene concentration was observed. This was in agreement with studies showing a positive dependence of isoprene unit formation on N fertilization (Mccullough and Kulman, 1991; Close et al., 2004). High N concentrations in leaves promoted photosynthetic activity, which increased the availability of assimilated carbon used to generate metabolites *via* the methylerythritol pyrophosphate (MEP) pathway (Ormeno and Fernandez, 2012). Two biosynthetic pathways contributed to the early steps in the production of plant terpenes. The first is the cytosolic mevalonic acid (MVA) pathway, which is involved in the biosynthesis of sesquiterpenes and triterpenes. The second, plastid-localized methylerythritol phosphate (MEP) pathway, is involved in the biosynthesis of monoterpenes, diterpenes, and tetraterpenes (Bouvier et al., 2005). Phytocannabinoids are synthesized from isoprenoid precursors combined with fatty acids (Dewick, 2002). However, the geranyl pyrophosphate necessary for the production of the terpenoid part of cannabinoids is predominantly (>98%) synthesized by the MEP pathway in plastids (Fellermeier et al., 2001). Because limonene, β-myrcene, and the terpenoid part of THCA are synthesized *via* the same MEP pathway and exhibit a concentration response in medical cannabis flowers opposite to that from addition of AAs to the nutrient solution, which increases N levels, it can be concluded that the biosynthesis of the ketide (fatty acid) part of the THCA molecule may be affected (Tedesco and Duerr, 1989). However, further research will be needed to draw relevant conclusions.

## Conclusion

This study investigated the effects of amino acid supplementation and two different nutritional cycles (systems) on medical cannabis growth. The exact relationship between the content of secondary metabolites and the nutritional supplements remains unclear. This connection is complex and involves several parameters, including nutrient availability, biosynthetic conditions, and physiological signals. The amino acid-based nutritional supplement significantly increased the nitrogen and sulfur content and reduced the accumulation of calcium and iron in both cycles throughout the plant. It caused earlier maturation in plants as reflected in the THCA concentration in the drain-to-waste cycle and reduced the CBNA content in flowers. Furthermore, in both nutritional cycles, it significantly increased the content of monoterpenes, limonene and β-myrcene. When comparing the nutritional cycles of treatments with the amino acid supplement, it can be seen that a significantly higher content of nitrogen and sulfur was achieved in the recirculation cycle, but a lower content of calcium in the whole plant. In the drain-to-waste cycle, medical cannabis plants matured about a month earlier, based on THCA concentration, but at the expense of half-maximal THCA yield in flowers and significantly lower concentrations of limonene and β-myrcene than with the recirculation cycle. This study clearly shows the advantages and disadvantages of the amino acid-based biostimulant and of the different nutritional cycles. In the recirculation cycle, higher yields of secondary metabolites were achieved with much lower total nutrient consumption, but over a more extended time. On the contrary, the drain-to-waste cycle allowed better control of the nutrient solution, stable supply of accurate nutrient concentration, and accelerated plant ripening, but with higher fertilizer consumption and lower overall yield of secondary metabolites. This study examined a high-yield THCA variety classified as chemotype I grown hydroponically in Euro Pebbles (expanded clay) medium. Therefore, it would be interesting to carry out these studies on cannabis varieties of different chemotypes.

## Data Availability Statement

The raw data supporting the conclusions of this article will be made available by the authors, without undue reservation.

## Author Contributions

MM designed the study, wrote the manuscript, controlled the cultivation scheme, and performed physiological, chemical, and data analyses. JV designed the study, controlled the cultivation scheme, and performed physiological and chemical analyses. LP and AJ performed chemical analyses. ZK controlled the cultivation scheme. PK supervised the study. PT designed and supervised the study. All authors contributed to the article and approved the submitted version.

## Funding

This study was supported by the European Regional Development Fund (project NUTRISK no. CZ.02.1.01/0.0/0.0/16_019/0000845).

## Conflict of Interest

The authors declare that the research was conducted in the absence of any commercial or financial relationships that could be construed as a potential conflict of interest.

## Publisher’s Note

All claims expressed in this article are solely those of the authors and do not necessarily represent those of their affiliated organizations, or those of the publisher, the editors and the reviewers. Any product that may be evaluated in this article, or claim that may be made by its manufacturer, is not guaranteed or endorsed by the publisher.
